# Seasonality of deaths with respect to age and cause in Chitral District Pakistan

**DOI:** 10.1371/journal.pone.0225994

**Published:** 2019-12-06

**Authors:** Muhammad Asif, Khalid Nawaz, Zafar Zaheer, Helene Thygesen, Amani Abu-Shaheen, Muhammad Riaz

**Affiliations:** 1 Department of Statistics, University of Malakand, Dir (L), Khyber Pakhtunkhwa, Pakistan; 2 Department of Statistics, Shaheed Banazir University, Sheringal, Pakistan; 3 Institute of Management Sciences, Peshawar, Khyber Pakhtunkhwa, Pakistan; 4 Department of Conservation, Hamilton, New Zealand; 5 Research Centre, King Fahad Medical City, Riyadh, Saudi Arabia; UCIBIO-REQUIMTE, Faculty of Pharmacy, University of Porto, PORTUGAL

## Abstract

**Background:**

Seasonal variability in mortality has been studied in various regions globally. Proper evaluation of seasonally fluctuating mortality is important to establish effective public health measures. We investigated the overall, age-specific, and cause-specific seasonality of deaths in Chitral District in Pakistan.

**Method:**

Data on 2577 deaths were provided by the Agha Khan Health Support Program. Seasonal mortality patterns concerning age and causes were examined using the X-12 ARIMA pseudo-additive decomposition method.

**Results:**

Of the total deceased, 59.6% were males. The proportion of deceased males was significantly higher than the female (40.4%, p< 0.001). The average age at death was 57.7 years (SD = 28.7). On average, approximately 43 deaths occurred each month. More than 10% of the deaths occurred in children less than 5-years-of-age. Among all the causes of death, the most frequent was cardiovascular disease (n = 666, 25.8%) followed by respiratory disease (n = 482, 18.7%). Significant seasonality in the overall deaths was evident, with a peak in winter. Deaths in people ≥ 55-years-of-age were significantly seasonal and peaked in winter. Deaths due to cardiovascular, respiratory, and kidney related diseases were also significantly seasonal with winter peaks. Further, deaths due to external causes were significantly seasonal with summer peak.

**Conclusion:**

In the winter season, all-cause, except external, and age-specific mortality peaks in Chitral District, Pakistan. Deaths due to external causes and cardiovascular, respiratory, and kidney related diseases were significant seasonal effects.

## Introduction

Proper evaluation of seasonally fluctuating factors, including atmospheric conditions, is important to establish effective public health measures. For example, human activity, seasonal variability in human immune system function, seasonal variations in vitamin D levels, seasonality of melatonin, and pathogen infectivity have explained the seasonality of directly transmitted infectious diseases [1]. Public awareness about the role of environmental factors (pollution, cold, infection and so on) or other factors such as indoor activity and vitamin D intake could be used to improve prevention measures and educational strategies [1]. The effect of seasons on mortality of humans has been known from the time of Hippocrates. In his essay “On Airs, Waters, and Places”, he wrote “Whoever wishes to investigate medicine properly, should proceed thus: in the first place to consider the seasons of the year, and what effects each of them produces for they are not at all alike, but differ much from themselves in regard to their changes” [2].

Seasonal variability in death rates is well-known in various regions of the world [3–14]. Many studies have been conducted to investigate and describe the influences of season on mortality from various perspectives, including meteorological, geographical, and socioeconomic variables [3]. One study [4] found a seasonal effect on deaths as a function of humidity and rainfall. Another study [5] reported lower death rates in cooler regions compared to warmer regions. Another study [6] that investigated a population in Moscow, Russia, reported lower vulnerability to heighted deaths in winter as compared to other western countries. Another study [7] reported that a Scottish population experienced 30% more deaths in winter than in summer with respiratory, coronary artery, and cerebrovascular diseases as the leading causes of deaths. The authors further reported that when the average weekly temperature fell one degree Celsius, there was a 1% rise in number of deaths in the following week. Yet another study [8] investigated the number of deaths in Barcelona and reported the highest and lowest number of deaths at the beginning of February and August, respectively. Similarly, in the Netherlands, deaths peaked in winter with cardiovascular and respiratory diseases as the lead causes [9]. A similar peak in the winter was reported from Canada [10]. A study from Japan reported that Japanese individuals are more prone to die in the winter months with respiratory disease, accidents, parasitic and infectious diseases, digestive diseases, and cerebrovascular diseases as the major causes of death [11]. In Burkina Faso, West Africa, increased deaths in winter were documented from all causes and cardiovascular diseases in particular [12]. Similarly, in Emilia-Romagna, Italy, people 80-89-years-of-age were reportedly more prone to die in the winter months than in the summer [13]. In the United States, seasonal variation in mortality involves increased numbers of elderly individuals and decreased numbers of young individuals dying in the in the winter months [14]. Finally and similarly, a study concluded that seasonality of deaths increased with age with differences in gender (men being more susceptible to die in winter) [15].

Knowledge of the seasonality patterns of deaths and the geographical and demographic characteristics is crucial for recognizing high-risk groups and setting appropriate and management strategies. No such information exists in Pakistan. This South Asian country features varied climatic settings. It experiences four seasons: a cool, dry winter starts from December and continues through the middle of March; a hot, dry spring persists from March through May; Southwest monsoons are a hallmark of the rainy summer season from June until September; finally, there is an autumn season during October and November. This variation results in a range of extreme weather events, including storms, floods, blizzards, and droughts accompanied by health complications in concert with variable rates of morbidity and mortality through the year [16].

In literature, no such study is available that investigates seasonality of deaths by causes and age, in Pakistan. The data will be important in terms of public health epidemiology.

## Material and methods

The records of the Agha Khan Health Support Program (AKHSP) for all deaths from January 2011 to December 2015 were reviewed by a member of their staff and anonymized data on 2577 deaths were provided for this analysis. AKHSP is a non-government organization providing basic health facilities to the people of Chitral District. The district is situated in the north-west Pakistan in the Hindukush Mountains. It is the largest district in the province of Khyber Pakhtunkhwa, and covers the area of 14,850 km^2^. The population is approximately 447,362 [17, 18]. According to the Koppen-Geiger climate classification scheme, the climate can be considered as BSk (Arid-Steppe-Cold) [19]. The winter is very cold and harsh, and the summer season from May to September is pleasant [20].

Whenever a death occurs, the local government dispatches a lady health visitor (LHV), a public health professional who advises and assists the residents in health related issues. Additionally, an AKHSP community health worker visits the house of the deceased and collects the following information from a member of the family: name of the deceased, name of father/ husband, gender, date of birth, date of death, immediate cause of death, and history of diseases. In cases where the exact date of birth is not known then June 15 of the year of birth is taken as the date of birth.

In the present study, the age of the deceased was determined from the date of birth and date of death and grouped into (≤ 4, 4–14, 14–24, 24–34, 34–44, 44–54, 54–64, 64–74, 74–84, 84–94, and 94+ years) to examine seasonality by age. Immediate causes of death were grouped into cardiovascular disease, respiratory disease, cancer, infection, kidney disease, external (deaths due to cocaine abuse, electric shock, gunshot, head injury, home accident, motor vehicle accident, murder, homicide, and poisoning suicide), and other causes. Ethical approval for the study was obtained from the Department of Biotechnology, University of Malakand Ethical Committee. To visualize the occurrence of deaths due to various causes, the average number of deaths that occurred over a 5-year period was graphed according to the aforementioned age groups. The seasonal indices of deaths for overall data, age group 55–65 years, and ≥ 65-years-of-age were determined and displayed by the months of the year.

To examine variation in patterns of mortality by age and cause of death, we computed average deaths per month categorized by ‘age’ and cause’. The time series data of deaths were available, and we were interested in estimating seasonal indices (seasonal effects on deaths). For this, we used the X-12 ARIMA procedure [21], developed by the United States Census Bureau. The X-12 census method-II is the most widely used method for decomposition. This is based on the basic seasonal adjustment procedure (X-11 ARIMA) that decomposes a monthly or quarterly time series into a trend component, seasonal component, and residual component. In this kind of decomposition, the seasonally adjusted series is obtained by dividing the original series by the estimated seasonal component. The decomposition is also performed by decomposing the series into a sum of trend, seasonal, and residual components and subtracting the estimated seasonal component to obtain the seasonally adjusted series. These adjustments are multiplicative and additive, respectively. Another adjustment, the pseudo-additive decomposition, is a mix of the two stated decompositions; it is useful for series with periodically small or zero values [21]. We have used X-12 ARIMA with the pseudo-additive method of decomposition with the robust estimation of coefficients, implemented in MS-Excel by Spider Financial software [22]. Compared with X-11 ARIMA, X-12 ARIMA has several advantages, which include the novel diagnoses of the quality and stability of the adjustment, and extensive range of time series modelling and model selection capabilities for linear regression models with ARIMA errors [21]. The method allows gradual changes in the seasonal component over time and estimates trend values, including the end points. Using the above method, the proportionate-change (PC) of indices for deaths can be determined as:

$$PC=\frac{{max}_{i=1,2,\ldots .12}\{\sum_{j=1}^{n-1}s(t+12j)\}}{{min}_{i=1,2,\ldots .12}\{\sum_{j=0}^{n-1}s(t+12j)\}}-1$$

where the seasonal indices s*(t)* are calculated by the X-12 US census method described above, and *n* represents the number of years, for example, 5 years in this study. Higher values of PC indicate greater seasonality of deaths. A value of indices, *s(t)*, greater than unity indicates above average deaths, while a value below unity indicates below average deaths in the season. To assess the statistical significance of the seasonality, we used the non-parametric Kruskal-Wallis test. For all analyses, results were considered statistically significant if p<0.05.

## Results

**Fig 1:**
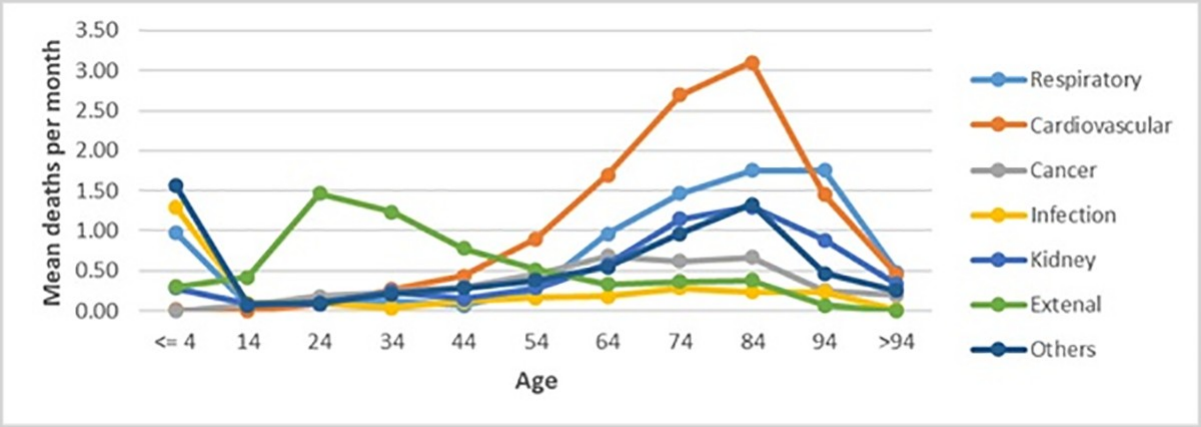
Cause-specific average number of deaths per month grouped by age.

From the total sample, 59.6% were males. The proportion of males was significantly higher than the proportion of females (40.4%, p<0.001). The average age at death was 57.7 years with a standard deviation of 28.7. On average, approximately 43 deaths per month occurred. More than 10% of deaths occurred in children under 5-years-of-age. Further, in the data highest proportions of deaths were caused by cardiovascular disease (n = 666, 25.8%) followed by respiratory disease (n = 482, 18.7%). Other causes, external, kidney disease, and infection were responsible for 371(14.4%), 352(13.7%), 322 (12.5%), and 165 (6.4%) of the total deaths, respectively. Table 1 shows the average number of deaths per month during the study period, grouped by age and causes. Fig 1 provides a graphical representation of the results in Table 1 and depicts the variation in average deaths per month with respect to age groups and morbidity.

**Table 1:** Average number of deaths per month grouped by causes and age.

Age groups, years	Respiratory	Cardiovascular	Cancer	Infection	Kidney	External	Others	All-Causes
**≤ 4**	0.98	0.02	0.00	1.30	0.28	0.30	1.57	**4.45**
**4–14**	0.07	0.00	0.07	0.10	0.08	0.42	0.07	**0.80**
**14–24**	0.12	0.08	0.18	0.08	0.08	1.47	0.10	**2.12**
**24–34**	0.12	0.27	0.23	0.03	0.23	1.23	0.22	**2.33**
**34–44**	0.07	0.43	0.30	0.12	0.15	0.78	0.28	**2.13**
**44–54**	0.27	0.90	0.45	0.17	0.28	0.52	0.38	**2.97**
**54–64**	0.97	1.70	0.68	0.18	0.58	0.33	0.55	**5.00**
**64–74**	1.47	2.70	0.62	0.28	1.15	0.37	0.97	**7.55**
**74–84**	1.75	3.10	0.67	0.23	1.30	0.38	1.33	**8.77**
**84–94**	1.75	1.45	0.25	0.23	0.88	0.07	0.47	**5.10**
**94+**	0.48	0.45	0.20	0.02	0.33	0.00	0.25	**1.73**
**All-Ages**	**8.03**	**11.10**	**3.65**	**2.75**	**5.37**	**5.87**	**6.18**	**42.95**

Of all the causes, most deaths occurred before the age of 4 years and after 54-years-of-age, with the exception of external causes, where the majority of deaths occurred for those aged 14–34 years.

### Seasonality of deaths in overall data

**Fig 2:**
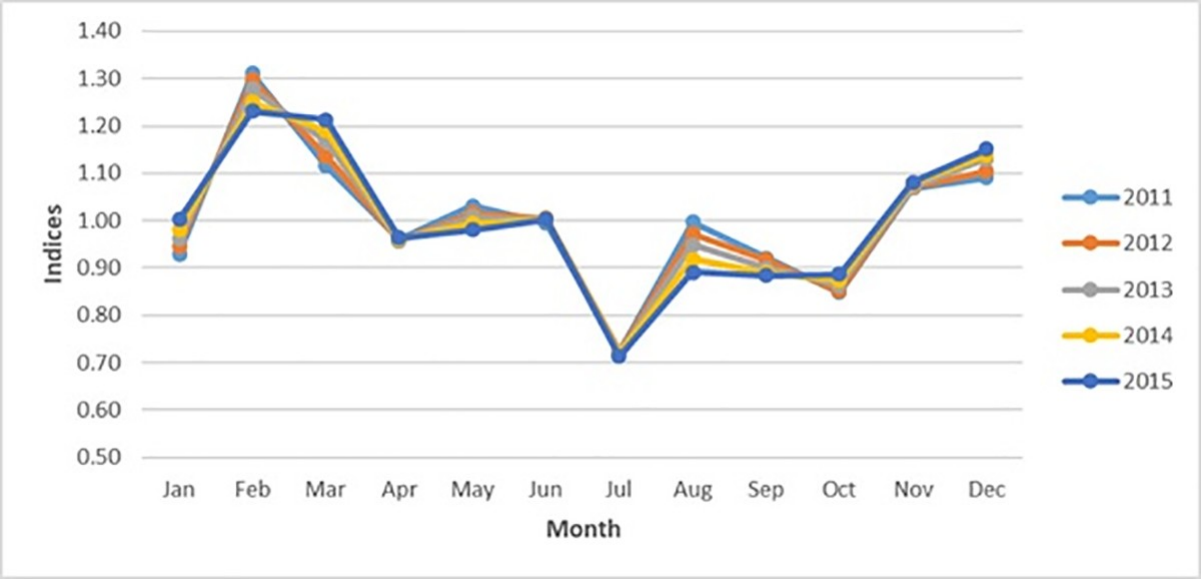
Seasonal indices pattern for the overall data categorized by year.

Significant seasonality was observed in the overall data with a winter peak. Fig 2 plots seasonal indices for each month grouped by years. The PC of the seasonality of 0.77 means that 77% more deaths occurred in winter as compared to summer.

### Seasonality of deaths by age

**Fig 3:**
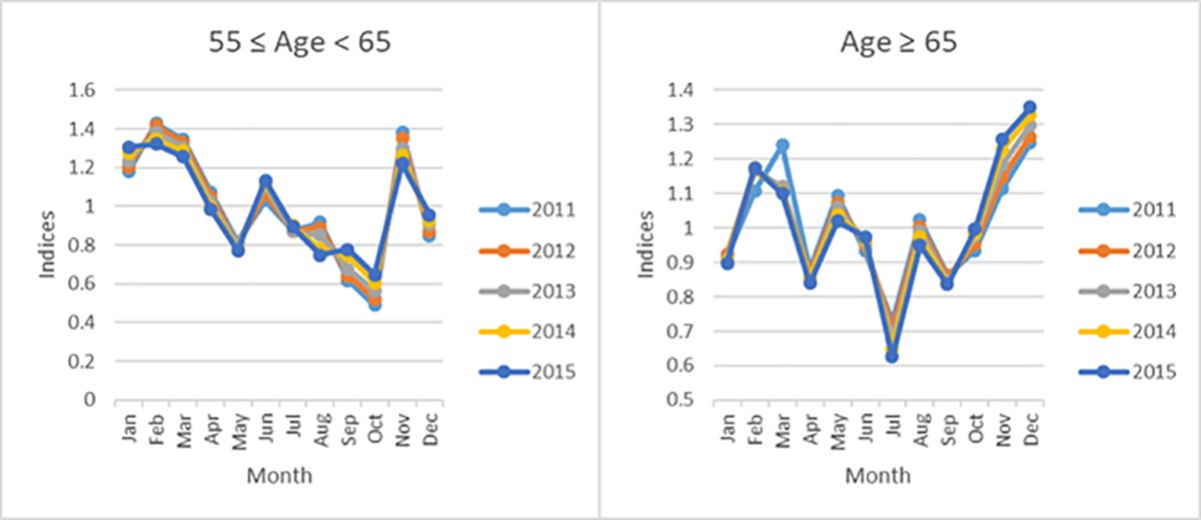
Monthly seasonal indices for the 55–65 and ≥ 65 years-of-age groups categorized by year.

A significant seasonal effect on deaths for individuals ≥ 55 years-of-age was apparent. For example, individuals between 55 and 65 years-of-age were more prone to die in winter compared to the summer (PC = 1.44). Similarly, individuals ≥ 65 years-of-age displayed a significant seasonal pattern dominated by winter (PC = 0.915). Fig 3 provides a graphical presentation of the seasonal pattern of the deaths for the 55–65 and ≥65-years-of-age groups

### Seasonality of deaths by cause

**Fig 4:**
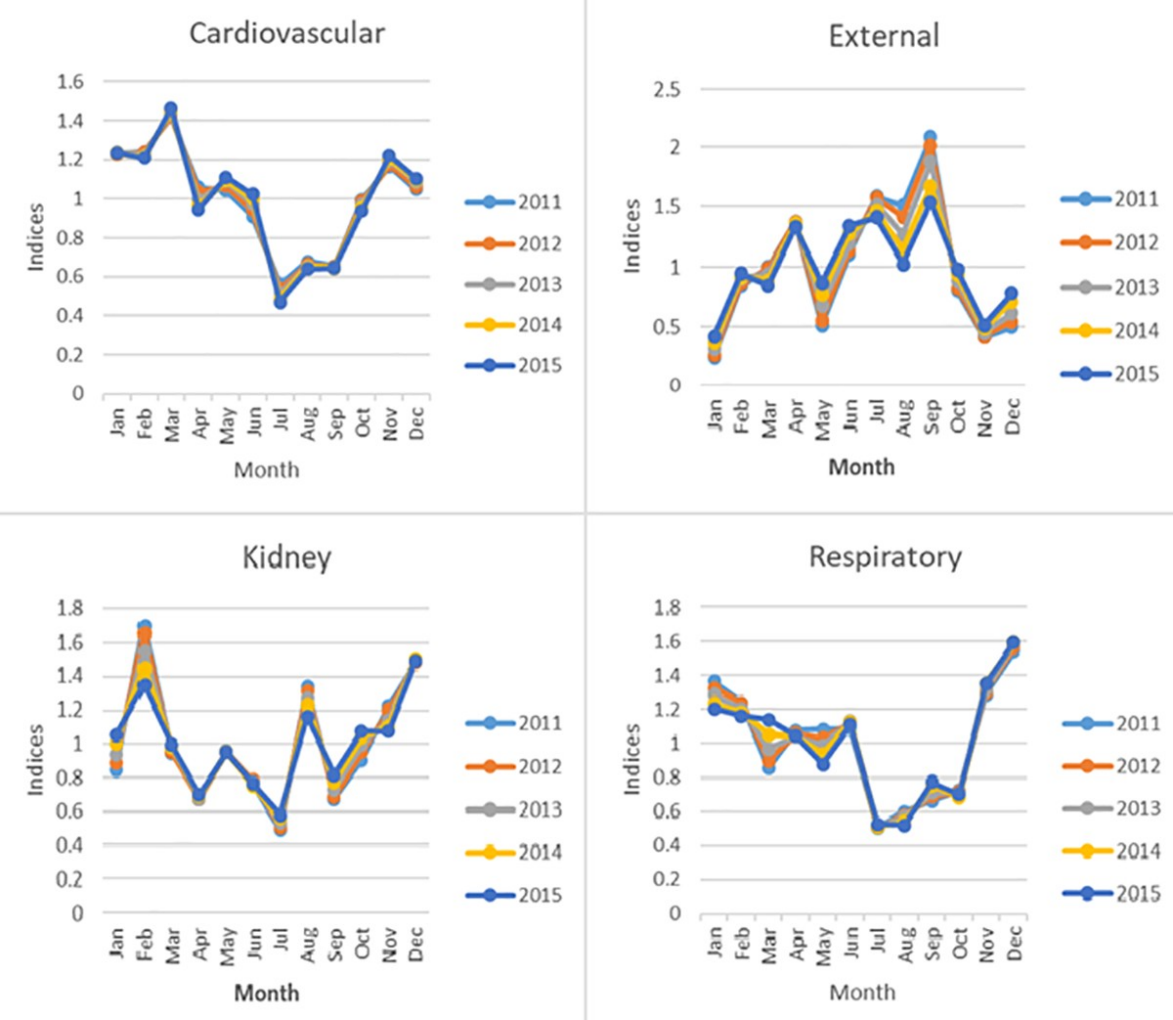
Monthly seasonal indices of deaths by causes grouped by year.

Table 2 presents the PC of mortality by causes. Cardiovascular, kidney, and respiratory related diseases, as well as external causes of deaths showed significant seasonality. Cardiovascular disease was the leading cause of death (25.8%), with a peak in winter and a trough in summer. Similar pattern of seasonality were observed in kidneys and respirateory related diseases. In contrast, deaths due to external causes peaked in summer and troughed in winter. The difference in monthly seasonal indices are depicted in Fig 4. Further, it is worth mentioning that significant seasonality in external causes were observed only when the monthly data transformed into quarterly data.

**Table 2:** Measures of seasonality by causes.

Cause*	Percentage	Trough, SI(Month)	Peak, SI(Month)	PC^a^
Cancer	8.5%	3.00 (Jan)	7.36 (Feb)	1.44
Cardiovascular	25.8%	2.57 (Jul)	7.19 (Mar)	1.79
External	13.7%	1.61 (Jan)	9.48 (Sep)	4.87
Infection	6.4%	2.28 (Aug)	9.83 (Mar)	3.32
Kidney	12.5%	2.66 (Jul)	7.69 (Feb)	1.88
Respiratory	18.7%	2.57 (Jul)	7.89 (Dec)	2.06
Others	14.4%	3.72 (Aug)	6.35 (Feb)	0.70

^**a**^ Proportionate Change

* Kruskal-Wallis test showed statistically significant seasonality for cardiovascular, external causes, kidney and respiratory related diseases, p-values<0.05

## Discussion

This paper investigated all-cause and age-specific mortality associated with seasons of the year in Chitral District, Pakistan. In this study, deaths due to cardiovascular, kidney and respiratory related diseases, as well as external causes were seasonal. The effects on mortality vary among the age groups.

More deaths occurred in February with the fewest in July. Seasonality of deaths in people ≥ 54-years-of-age was significant with winter peaks. The remaining age groups did not show statistically significant seasonality of deaths. Furthermore, seasonality was observed in deaths due to external causes. It is obvious from Table 1 that higher average number of deaths per month due to external causes occurred in the younger age group (14–34 years). This may reflect the fact that majority of young people ride motor-bikes and bicycles in the region, with less safety measures, such as not wearing helmets, while riding during the summer. In addition, there are no adequate road systems and existing roads are in need of repair, which may have contributed to fatal accidents. Also, deaths due to external causes peaked during the hot summer months, when people spend more time outdoors, and deaths from infectious diseases are more prevalent in spring. Deaths due to external causes also included deaths due to cocaine abuse, electric shock, gunshot, head injury, home accident, murder, homicide, and self-inflicted poisoning.

Among the seven causes of deaths, cardiovascular, respiratory, and kidney related diseases were found to be seasonal with winter peaks. The potential underlying mechanisms and factors could be pronounced variations in ambient temperature and milder climates in general [20, 23], lack of preparation for extreme weather variations, air pollution levels, and concurrent influenza [24]. However, we lacked data on weather conditions and environmental factors during the study period; this may be investigated further in a rigorously conducted study. Our findings are similar to that reported in the studies (worldwide) investigating seasonal effects on mortality with worst scenarios in winter for cardiovascular diseases and older age groups [7, 8, 11, 12, 13, 14, 25, 26]. In addition, a few studies have described that the increased risk of mortality due to cardiovascular and respiratory diseases, external causes, and gastrointestinal infections is associated with elevated heat [27, 28, 29, 30]. It is worth mentioning that the heat-related mortality due to cardiovascular diseases reported in these studies are due to the increased stress on the circulatory system [29], whereas the majority of the studies documented increased risk of mortality due to cardiovascular disease in winter. Moreover, the winter climate of this region in Pakistan is not much different from the climate of Northern Europe. Therefore, the findings of our study are in line with the results of studies from countries of Northern Europe, particularly, data from England, Wales, Netherlands, Denmark, and Portugal showing that cardiovascular mortality was lowest at the end of August [27]. Studies based on data from Germany, Norway, and Ireland have also indicated similar findings for all-cause and cardiovascular disease related mortality [31, 32]. A study of the underlying mechanisms and factors associated with seasonality of cardiovascular disease mortality reported that cold temperatures during the winter months were associated with increased sympathetic nervous system activation and catecholamine secretion as well as, vitamin D deficiency, high serum cholesterol, lack of physical activity, increase risk of coagulation, blood pressure related hormones such as arginine vasopressin, norepinephrine, and angiotensin II, respiratory infection, age, and sex [24].

Our analyses showed that people in Chitral District, Pakistan are at high-risk of death during the winter season compared to the summer season. The majority of people in the region have a very low standard of living and reside in low quality houses; the higher risk of winter mortality may be reduced by improving the housing conditions [33, 34]. Work load reduction (e.g., vacations), safety measures, improving the conditions of roads and buildings, and a better communication system in the region may help in reducing the higher risk of winter mortality. Policy makers and public health departments need to evaluate the risk and consider providing adequate emergency services to the vulnerable in the region. Government and non-governmental organizations could run poverty reduction programs to support the poor in the region and improve their quality of life. Examples include providing funding or low-interest loans for developmental projects in education, agriculture, livestock, housing; establishing small scale industrial units; boosting household income through a monthly package for poor families; and providing support in times of household economic crises.

One strength of this study was our use of the X-12 ARIMA with a time series analysis of seasonality in the time domain developed by the US Census Bureau. Some categories had zero values, so the pseudo-additive decomposition without prior adjustment was used to assess the seasonality of deaths. Moreover, the data for this analysis was from a reliable source, AKHSP, which provides a wide variety of health services in the region to improve health outcomes. However, our data is limited by unavailability of sociodemographic factors to adjust the results. In addition, the data is from just one region of Pakistan with no data from other regions to perform comparisons, and there is a pronounced variation in the climate of Pakistan. Nonetheless, the results obtained in this study are consistent with published results worldwide.

## Conclusions

People in Chitral District of Pakistan are at high risk of death during the winter season as compared to the summer season. Seasonality of deaths in people ≥ 54-years-of-age was significant, exhibiting winter peaks. Cardiovascular, respiratory and kidney diseases, as well as external causes of deaths showed significant seasonality. This fluctuation of mortality throughout the year could be attributed to the severity of weather, particularly excess cold. However, other factors such as socioeconomic and demographic variables might also be responsible for the fluctuation of mortality. Further studies are required to investigate this.

## Supporting information

S1 Data Deceased data with variables ‘Age’, ‘Month’, ‘Year’ and ‘Cause’. (XLSX)

S2 Data Seasonal indices grouped by age and cause. (XLSX)
